# P47 From commensal to culprit: the genetic diversity driving *Staphylococcus epidermidis* infections

**DOI:** 10.1093/jacamr/dlaf118.054

**Published:** 2025-07-14

**Authors:** William Martin, Heather Felgate, Mark Webber

**Affiliations:** University of East Anglia, Norwich, UK; Quadram Institute, Norwich, UK; Centre for Microbial Interactions, Norwich, UK; University of East Anglia, Norwich, UK; Quadram Institute, Norwich, UK; Centre for Microbial Interactions, Norwich, UK; University of East Anglia, Norwich, UK; Quadram Institute, Norwich, UK; Centre for Microbial Interactions, Norwich, UK

## Abstract

**Background:**

*Staphylococcus epidermidis* is one of the most isolated micro-organisms from clinical samples. Often considered a skin commensal, *S. epidermidis* infection can be a significant cause of morbidity and mortality, primarily associated with medical device-related infections such as Periprosthetic Joint Infections (PJI), Late Onset Neonatal Sepsis (LOS), both of which are becoming increasingly prevalent. Rising use of prosthetic medical devices and increasing antimicrobial resistance (AMR) complicate treatment. Increased usage of prosthetic medical devices and increased antimicrobial resistance presents significant challenge to treating *S. epidermidis* infection. There is limited understanding of how a commensal *S. epidermidis* goes on to cause infection, although we know that the genome of *S. epidermidis* contains a wide diversity of genes associated with AMR, biofilms and metal tolerance, which are thought to contribute to *S. epidermidis* ability to persist on medical devices and host evade immune responses.

**Objectives:**

This research aims to uncover the population structure of *S. epidermidis* and elucidate its genotype-phenotype associations with regards to AMR, biofilm formation and metal tolerance.

**Methods:**

A collection of clinical and non-clinical *S. epidermidis* isolates (*n*=378) from UK and Germany were subjected to WGS (Illumina and MinION). A phylogenetic tree of core and pan-genomes constructed using ROARY, distinguished phylogenetic groups with various AMR and Biofilm-associated genes mapped to the tree. The isolates MIC against 5 common antimicrobials were tested. Biofilm formation and metal tolerance against Copper, Zinc and Cobalt, and cross-tolerance between heavy metals and anti-microbials were also investigated.

**Results:**

*S. epidermidis* is genetically highly diverse group of Staphylococci. A phylogenetic tree based on core gene presence and absences shows various distinct groups of *S. epidermidis*, of which some were associated with PJI and nosocomial sources. Of these more clinically relevant groups, one was associated with the *ica* gene, however, were shown to be poorer biofilmers. On testing isolates from across the tree to various metal concentrations did not cause cross tolerance to antimicrobials or affect biofilm formation. Using scoary, it was shown that metal tolerance genes were dispersed across all groups of *S. epidermidis,* with specific genes more prevalent in particular groups. For resistance genes to copper (*copB* and *copC*), cadmium (*cadA*) and arsenic (*ars*) were highly associated with specific groups.

**Conclusions:**

This research has uncovered significant diversity in the phylogeny and population structure of *S. epidermidis*. The findings have emphasized the complex nature of *S. epidermidis* infection and its phenotype-genotype associations. Mechanisms of biofilm formation, AMR, and metal tolerance are not consistent within the species. Being able to better identify genes associated with infections from *S. epidermidis,* may allow insights into the development of better diagnostic techniques and novel therapeutic agents.

